# In Vitro Characterization of Motor Neurons and Purkinje Cells Differentiated from Induced Pluripotent Stem Cells Generated from Patients with Autosomal Recessive Spastic Ataxia of Charlevoix-Saguenay

**DOI:** 10.1155/2023/1496597

**Published:** 2023-04-15

**Authors:** Aurélie Louit, Marie-Josée Beaudet, Mathieu Blais, François Gros-Louis, Nicolas Dupré, François Berthod

**Affiliations:** ^1^LOEX, Centre de recherche du CHU de Québec-Université Laval, Quebec City, Quebec, Canada; ^2^Department of Surgery, Faculty of Medicine, Université Laval, Quebec City, Quebec, Canada; ^3^Department of Medicine, Faculty of Medicine, Université Laval, Quebec City, Quebec, Canada

## Abstract

Autosomal recessive spastic ataxia of Charlevoix-Saguenay (ARSACS) is an early-onset neurodegenerative disease mainly characterized by spasticity in the lower limbs and poor muscle control. The disease is caused by mutations in the *SACS* gene leading in most cases to a loss of function of the sacsin protein, which is highly expressed in motor neurons and Purkinje cells. To investigate the impact of the mutated sacsin protein in these cells *in vitro*, induced pluripotent stem cell- (iPSC-) derived motor neurons and iPSC-derived Purkinje cells were generated from three ARSACS patients. Both types of iPSC-derived neurons expressed the characteristic neuronal markers *β*3-tubulin, neurofilaments M and H, as well as specific markers like Islet-1 for motor neurons, and parvalbumin or calbindin for Purkinje cells. Compared to controls, iPSC-derived mutated *SACS* neurons expressed lower amounts of sacsin. In addition, characteristic neurofilament aggregates were detected along the neurites of both iPSC-derived neurons. These results indicate that it is possible to recapitulate *in vitro*, at least in part, the ARSACS pathological signature in vitro using patient-derived motor neurons and Purkinje cells differentiated from iPSCs. Such an *in vitro* personalized model of the disease could be useful for the screening of new drugs for the treatment of ARSACS.

## 1. Introduction

Autosomal recessive spastic ataxia of Charlevoix-Saguenay (ARSACS) is a childhood-onset hereditary neurodegenerative disease. ARSACS is considered the second most frequent ataxia after the Friedreich ataxia [1]. Described for the first time in the 1970s [2] with a carrier prevalence of 1/22 in the Charlevoix-Saguenay region of the Quebec Province, ARSACS is now found in more than twenty countries worldwide [3, 4]. Different causative mutations in the *SACS* gene are linked to the disease. To date, more than 200 mutations have been identified in this gene [5]. The *SACS* gene encodes the protein sacsin, highly expressed in Purkinje cells and motor neurons (MNs), which is involved in molecular chaperoning, mitochondrial transport and integrity, and neurofilament assembly [6]. Sacsin knockout mice recapitulated at least in part histopathological and neurological features of ARSACS, pointing out to a sacsin loss-of-function disease mechanism [7]. ARSACS is characterized by cerebellar, pyramidal, and neuropathic involvement and remains incurable [8]. Noteworthy, research on ARSACS patient fibroblasts and mice with decreased *SACS* gene expression showed mitochondrial abnormalities, with hyperfused mitochondria and a reduction in Drp1-mediated mitochondrial fission [9–11]. Mitochondria autophagy was studied on a commercial neuron-like cell line, and an alteration of this mechanism was discovered [12]. Studies conducted using ARSACS mouse models also indicated an alteration in the organization of the intermediate filament network with an accumulation of neurofilaments [7, 13]. Although the actual ARSACS animal models are still valuable to investigate the role of sacsin in the pathology of the disease, more reliable human-derived cellular models need to be generated to better understand the underlying pathophysiology of ARSACS in specific cell types. To overcome the lack of human tissue accessibility, interest has now focused on the differentiation of patient-derived induced pluripotent stem cells (iPSCs) into cerebellar cells such as Purkinje cells or granule cells [14–16]. A cerebellum organoid model has also been developed to recapitulate the complexity of cerebellar tissue [17]. However, organoids sometimes cause some reproducibility issues [18–20].

In this paper, we propose reproducible methods to differentiate ARSACS patient-derived iPSCs into MNs and Purkinje cells. We also show for the first time the detection of ARSACS pathological features in patient-derived neuronal cells to confirm the pathological effects caused by the observed impairment of sacsin in mouse models or patient-derived nonneuronal cells.

## 2. Materials and Methods

### 2.1. Cell Culture

This study was approved by the ethical committee of the CHU de Québec-Université Laval (#2019-4444). The human cells used in these experiments were obtained following informed consent from the donors.

As previously described [21], healthy human dermal fibroblasts were isolated from skin biopsies after breast reductive surgery and cultured in DMEM-F12 medium supplemented with 10% fetal calf serum (FCS; Wisent, St-Bruno, Canada) and 25 *μ*g/ml of penicillin/gentamycin (MilliporeSigma, Oakville, Canada). Human epineural fibroblasts were obtained after peripheral nerve surgery from a small piece of nerve epineurium and cultured in DMEM-F12 supplemented with 20% FCS and antibiotics (100 U/ml penicillin and 25 *μ*g/ml gentamycin).

### 2.2. Differentiation of iPSCs into MNs

The iPSCs were obtained from the iPS-Quebec platform of the CHU de Québec-Université Laval Research Center. They were derived from 3 healthy human lymphoblast cell lines 522-617 (C1), 522-1839 (C2), and 522-25651 (C3) and from 3 ARSACS human skin fibroblast cell lines FARSF56-7 (A1), FARSM50-5 (A2), and SK.01AiPS (A3, a generous gift from Dr. Peter McPherson) (Table 1 in Supplementary materials). The iPSCs were reprogrammed with the CytoTune™-2.0 Sendai Reprogramming Kit (Invitrogen, Carlsbad, CA).

Differentiation of iPSCs into MNs was conducted following a protocol adapted from references [22, 23]: iPSCs were dissociated with StemPro Accutase (Thermo Fisher Scientific, Waltham, MA), combined with 5 *μ*M of the Rho kinase inhibitor Y-27632 (Abcam, Toronto, Canada), diluted in DMEM-F12 (3 : 1) medium added with 5 *μ*M Y-27632 without serum. The iPSCs were plated at a density of 90,000 cells/cm^2^ on Geltrex (1 : 200, Thermo Fisher Scientific) coated T75 flasks and were dissociated at confluency with StemPro Accutase with 5 *μ*M Y-27632. From day 0 to day 10 of differentiation, a basic supplemented medium was used and contains the following: DMEM-F12 : neurobasal A medium (3 : 1, vol : vol) (Thermo Fisher Scientific) supplemented with 1% N2 supplement (Thermo Fisher Scientific), 1% B-27 supplement minus vitamin A (Thermo Fisher Scientific), 10 *μ*M *β*-mercaptoethanol (Thermo Fisher Scientific), 50 *μ*g/ml ascorbic acid (MilliporeSigma), 5 *μ*M Y-27632, 1% L-alanyl-L-glutamine (Corning, New York, NY), 1% MEM nonessential amino acids (Thermo Fisher Scientific), 0.1% trace element A, 0.1% trace element B, 0.1% trace element C (Thermo Fisher Scientific), and antibiotics (100 U/ml penicillin and 25 *μ*g/ml gentamycin). From days 0 to 3 of differentiation, the basic supplemented medium also had 40 *μ*M SB431542 (MilliporeSigma), 0.2 *μ*M LDN193189 (MilliporeSigma), and 3 *μ*M CHIR99021 (MilliporeSigma) added. On days 2 and 3, the basic supplemented medium was additionally complemented with 0.88 nM retinoic acid (MilliporeSigma) and 500 nM SAG (Abcam). From day 4 to day 8, only 0.88 nM retinoic acid (MilliporeSigma) and 500 nM SAG (Abcam) were added to the basic supplemented medium. On days 9 and 10 of differentiation, the basic supplemented medium was only complemented with 10 *μ*M DAPT (Selleck Chemicals LLC, Houston, TX). At day 11, cells were passaged with StemPro Accutase added 5 *μ*M Y-27632 and seeded at 200,000 cell/cm^2^ on 50 *μ*g/ml poly-D-lysine (MilliporeSigma) treated plates if used for cell characterization or 285,000 cells/cm^2^ if seeded on the 3D sponge model. From day 11 until the end of the culture, the basic supplemented medium without *β*-mercaptoethanol was complemented with 20 ng/ml BDNF (Bon Opus Biosciences LLC, Millburn, NJ), 10 ng/ml GDNF (abm, Richmond, Canada). From day 13 to day 18 in 2D culture or until day 74 in 3D cultures, the basic supplemented medium was enriched with 10 ng/ml NT-3 (FroggaBio, Concord, Canada). DAPT was added only from days 9 to 12 and removed at day 13 in 2D culture but was present until day 18 in 3D cultures.

The purity of MNs was assessed on 3 control iPS cell lines at day 12 of differentiation. To quantify the number of islet1 and *β*3Tubulin-positive cells, 50,000 cells/cm^2^ were seeded in 24-well plates, and 6 wells were counted by immunocytochemistry, with 3 counts per well on randomly selected area (between 75 and 200 cells/area) using an Imager M2 stereo investigator (Carl Zeiss Microscopy). The results were expressed as the percentage of islet1 and *β*3Tubulin-positive cells in each of the 3 different cell lines normalized against iPSCs that do not express both markers.

### 2.3. Differentiation of iPSCs into Purkinje Cells

Differentiation of iPSCs into Purkinje cells was conducted following modifications of protocols established from [15, 16, 24–34]. The iPSCs were dissociated with StemPro Accutase (Thermo Fisher Scientific), combined with 5 *μ*M Y-27632 (Abcam), and diluted in DMEM-F12 medium added with 5 *μ*M Y-27632 without serum. The iPSCs were plated at a density of 90,000 cells/cm^2^ on Geltrex (1 : 200) coated T75 flasks and were dissociated at confluency with StemPro Accutase with 5 *μ*M Y-27632. From day 0 to day 11 of differentiation, cell culture basic medium contained DMEM-F12: neurobasal A medium (3 : 1) supplemented with 1% N2 supplement, 1% B-27 supplement minus vitamin A, 0.01 mM *β*-mercaptoethanol (Thermo Fisher Scientific), 50 *μ*g/ml ascorbic acid (MilliporeSigma), 5 *μ*M Y-27632, 1% L-alanyl-L-glutamine (Corning, New York, NY), 1% MEM nonessential amino acids (Thermo Fisher Scientific), 0.1% trace element A, 0.1% trace element B, 0.1% trace element C (Thermo Fisher Scientific), and antibiotics (100 U/ml penicillin and 25 *μ*g/ml gentamycin). From day 11 to the end, *β*-mercaptoethanol was removed from this basic medium. From days 0 to 4 of differentiation, 40 *μ*M SB431542 (MilliporeSigma), 0.2 *μ*M LDN193189 (MilliporeSigma), and 3 *μ*M CHIR99021 (MilliporeSigma) were added to the medium, but at day 4, SB431542 and LDN19318 were removed and CHIR99021 was removed at day 7. From day 2 to day 9 of differentiation, the medium was supplemented with 7 *μ*g/ml insulin and 35 ng/ml *β*FGF. At days 7 and 8, 10 *μ*M cyclopamine was added to the medium. From days 9 to 18 of differentiation, 10 *μ*M DAPT was added to the medium. At day 11, cells were dissociated with StemPro Accutase (Thermo Fisher Scientific), combined with 5 *μ*M Y-27632 (Abcam), diluted in DMEM-F12 medium without serum and containing 5 *μ*M Y-27632, and seeded at 400,000 cell/cm^2^ on 10 *μ*g/ml poly-D-lysine (MilliporeSigma) plates for cell characterization or 285,000 cells/cm^2^ over the 3D sponge model. At day 11, medium was added with 100 ng/ml NT-3 (FroggaBio) until day 19, 10 pg/ml GDNF until the end, and 1 nM urocortin until day 15 (R&D System, Oakville, Canada). From days 12 to 21 of differentiation, medium was supplemented with 30 nM T3 (3,3′,5-triiodo-L-thyronine sodium salt, MilliporeSigma). From days 13 to 18, 50 ng/ml FAIM2/LFG protein (Abcam) was added to the medium. From days 15 to 25 of differentiation, 1 nM corticotropin-releasing factor (CRF, Bio-Techne, Toronto, Canada) was added daily to the medium for a 12-hour period. From day 19 until the cell peeled off in the 2D experiment or to day 53 in the 3D experiment, the medium was supplemented with 20 ng/ml BDNF.

The purity of Purkinje cells was assessed on 3 control iPS cell lines at day 14 of differentiation. To quantify the number of KIRREL2 and *β*3Tubulin-positive cells, 50,000 cells/cm^2^ were seeded in 24-well plates, and 6 wells were counted by immunocytochemistry, with 3 counts per well on randomly selected area (between 75 and 200 cells/area) using an Imager M2 stereo investigator (Carl Zeiss Microscopy). The results were expressed as the percentage of KIRREL2 and *β*3Tubulin-positive cells in each of the 3 different cell lines, normalized against iPSCs that do not express both markers.

### 2.4. Differentiation of iPSCs into Schwann Cells

Differentiation of iPSCs into Schwann cells was performed as previously described [23, 35, 36]. Up to day 3, iPSCs were cultured in the same conditions than for MN differentiation (see above) to reach a neuronal precursor commitment. At day 3, cells were cultured in minimum essential medium (MEM, STEMCELL Technologies, Vancouver, Canada) with 1% N2 supplement, 1 mM *β*-mercaptoethanol, 5 mM Y-27632, 1% L-alanyl-L-glutamine, 1% MEM nonessential amino acids, 0.1% trace element A, 0.1% trace element B, 0.1% trace element C, and antibiotics (100 U/ml penicillin and 25 *μ*g/ml gentamycin). From day 4, *β*-mercaptoethanol was removed and 0.88 nM retinoic acid was added until day 6. At day 5, cells were passaged with StemPro Accutase, combined with 5 *μ*M Y-27632, diluted in DMEM-F12 medium added with 5 *μ*M Y-27632 without serum, seeded at 135,000 cell/cm^2^ on Geltrex (1 : 200) plates, and cultured in differentiation medium added from this day with 1% B-27 supplement but without vitamin A. At day 7, iPSCs were passaged again, seeded on 10 *μ*g/ml poly-D-lysine, and cultured in the same medium as day 5 but supplemented with 5 ng/ml PDGF-bb (Thermo Fisher Scientific), 10 ng/ml *β*FGF, 14 *μ*M forskolin (MilliporeSigma), and 192 ng/ml NRG1 (R&D System, Oakville, Canada). From day 9 until day 14, MEM was replaced by a mix 2 : 1 of DMEM-F12 : neurobasal A medium and 1% BSA-20% pH 7.4 (Bio Basic, Markham, Canada). At day 14, cells were passaged with StemPro Accutase, combined with 5 *μ*M Y-27632, diluted in DMEM-F12 medium added with 5 *μ*M Y-27632 without serum, and seeded at 95,000 cell/cm^2^ on 10 *μ*g/ml poly-D-lysine and 20 *μ*g/ml laminin (MilliporeSigma) coated plates. At day 21, cells were passaged and seeded at 285,000 cells/cm^2^ over the 3D sponge model to provide support for MNs.

### 2.5. Preparation of 3D Cell Culture Substrates

Collagen sponges were prepared as previously described [37, 38] but without chondroitin 4-6 sulphate. Briefly, type I and III bovine collagen (Symatese, Chaponost, France) and chitosan (Kemestrie, Sherbrooke, Canada) were dissolved in 0.1% acetic acid and mixed, and 0.5 ml of the mixture was poured into 12-well plates and frozen at -80°C for 1 h. The frozen plates were then lyophilized in a vacuum lyophilizer (Dura-Stop Microprocessor Controlled Tray Freeze-Dryer; FTS Systems, Stone Ridge, NY).

#### 2.5.1. Purkinje Cell Differentiation

To culture Purkinje cells in 3D, 210,000 dermal healthy human fibroblasts/cm^2^ were seeded onto the collagen/chitosan sponge and cultured for 2 weeks immersed in DMEM-F12 supplemented with 10% HyClone serum with 50 *μ*g/ml ascorbic acid and antibiotics (100 U/ml penicillin and 25 *μ*g/ml gentamycin). Then, the sponge was rinsed with DMEM-F12 and seeded with 285,000 Purkinje cells/cm^2^ at day 11 of differentiation. Then, samples were cultured in DMEM-F12 : neurobasal A medium (vol : vol) supplemented with 1% N2 supplement, 1% B-27 supplement minus vitamin A, 50 *μ*g/ml ascorbic acid, 5 *μ*M Y-27632, 1% L-alanyl-L-glutamine, 1% MEM nonessential amino acids, 0.1% trace element A, 0.1% trace element B, 0.1% trace element C, 1 ng/mL GDNF, 20 ng/mL BDNF, and antibiotics (100 U/ml penicillin and 25 *μ*g/ml gentamycin). iPS-derived Purkinje cells were differentiated for a total of 53 days.

#### 2.5.2. MN Differentiation

To culture the MNs in 3D, 210,000 dermal human fibroblasts/cm^2^ were seeded onto the collagen/chitosan sponge and cultured for 1 week. The sponge was flipped upside down and put at the air-liquid interface, and 100,000 human epineural fibroblasts/cm^2^ were seeded onto the sponge and cultured for one week with DMEM-F12 supplemented with 10% FCS and antibiotics (100 U/ml penicillin and 25 *μ*g/ml gentamycin). Sponge was rinsed, and 260,000 MNs/cm^2^ at day 11 of differentiation were seeded on the dermal fibroblast side. On day 13 of differentiation, the culture medium was composed of DMEM-F12 : neurobasal A medium (vol : vol), 1% N2 supplement, 1% B-27 supplement minus vitamin A, 50 *μ*g/ml ascorbic acid, 5 *μ*M Y-27632, 1% L-alanyl-L-glutamine, 1% MEM nonessential amino acids, 0.1% trace element A, 0.1% trace element B, 0.1% trace element C, 10 *μ*M DAPT, 10 ng/mL GDNF, 20 ng/mL BDNF, 10 ng/ml NT-3, and antibiotics (100 U/ml penicillin and 25 *μ*g/ml gentamycin). One week after the MN seeding, ARSACS or normal iPSC-derived Schwann cells were seeded on the same side as the epineural fibroblasts; DAPT was removed, and 10 ng/ml NGF was added until the end of the culture 8 weeks later (for a total of 74 days of iPS-derived MN differentiation).

### 2.6. Immunofluorescence

Cells cultured on plastic or on 3D substrates were fixed, respectively, 20 min and 1 h at 4°C with 4% paraformaldehyde and washed with cold PBS containing 0.3% Triton X-100 (Bio-Rad, Mississauga, Canada). 3D substrates were cut into 15 mm^2^ pieces or into 30 *μ*m cross-sections. Then, cells and 3D substrates were incubated 20 min at room temperature in an immunofluorescence staining buffer containing cold PBS, 0.3% Triton X-100 (Bio-Rad), and 5% serum. Cells and 3D substrates were incubated overnight at 4°C in the immunofluorescence staining buffer containing selected primary antibodies and were washed three times with PBS containing 0.3% Triton X-100. Cells and 3D substrate cross-sections were then incubated 1 h at room temperature in the immunofluorescence staining buffer containing selected secondary antibodies diluted (1 : 500). Finally, cells and 3D substrate cross-sections were washed with PBS -0.3% Triton X-100 and one last time with PBS before mounting with Fluoromount-G containing DAPI (Electron Microscopy Sciences, Hatfield, PA). Imaging was carried out using a LSI 700 confocal microscope with Zeiss Axio Imager (Carl Zeiss Microscopy, Jena, Germany).

The primary antibodies used were mouse anti-ß3Tub (1 : 500; BioLegend, San Diego, CA), chicken anti-neurofilament M 160 kDa (1 : 500; MilliporeSigma), mouse anti-neurofilament M 160 kDa (1 : 500; Abcam), rabbit anti-Islet-1 (1 : 1000; Abcam), rabbit anti-ChAT (1 : 1000; Millipore), rabbit anti-parvalbumin (1 : 200, Thermo Fisher Scientific), mouse anti-calbindin (1 : 100; Abcam), rabbit anti-PCP2 (1 : 500; Thermo Fisher Scientific), rabbit anti-KIRREL2 (1 : 500; Abcam), rabbit anti-Grid2 (1 : 500; Thermo Fisher), rabbit anti-sacsin (1 : 500; Abcam), and mouse anti-DRP1 (1 : 500; Abcam).

The secondary antibodies used were Alexa Fluor 647 donkey anti-rabbit (Invitrogen, Carlsbad, CA), Alexa Fluor 555 goat anti-mouse (Invitrogen), and Alexa Fluor 488 donkey anti-chicken (Jackson ImmunoResearch, West Grove, PA).

### 2.7. Western Blot Analysis

To extract total proteins from Purkinje cells cultured on 3D substrates, samples were lysed in 1x RIPA buffer (Abcam) and 1x Pierce protease and phosphatase inhibitor mini tablet (Thermo Fisher Scientific). The protein amount extracted from each Purkinje cell substrate was assessed with a Bio-Rad Protein Assay (Bio-Rad Laboratories Inc., Hercules, CA). An equal amount of lysates was loaded on a stacking gel and run in 8% or 10% size-fractioned SDS-polyacrylamide gel electrophoresis. Proteins were transferred to a polyvinylidene difluoride blotting membrane (Bio-Rad). Membranes were blocked in 5% nonfat dried milk containing 0.05% Tween 20 (VWR, Radnor, PA) and Tris-buffered saline (TBS) for 1 hour. Membranes were then probed overnight at 4°C with primary antibodies such as rabbit anti-sacsin (1 : 1000; Abcam), mouse anti-DRP1 (1 : 1000; Abcam), rabbit anti-vinculin (1 : 1000; Cell Signaling Technology, Whitby, Canada), and mouse anti-*β*-actin (1 : 1000; Abcam) diluted in the blocking solution. Membranes were washed in TBS-0.05% Tween 20 and incubated for 1 hour at room temperature with secondary antibodies such as goat anti-rabbit IgG-HRP (1 : 5000; Jackson ImmunoResearch Inc.) and goat anti-mouse IgG-HRP (1 : 5000; Jackson ImmunoResearch Inc.) diluted in blocking solution. Membranes were incubated with Amersham ECL Western Blotting Detection Reagent (GE Healthcare, Mississauga, Canada) to detect protein expression and were imaged with a Fusion Fx7 imager (Vilber Lourmat Sté, Collégien, France). The densitometric analyses of the bands were performed using ImageJ software (National Institutes of Health, Bethesda, MD). DRP1 protein expression was normalized to *β*-actin (42 kDa), but sacsin protein expression was normalized to vinculin (117 kDa) because of its high molecular weight (520 kDa).

### 2.8. Statistical Analysis

Statistical analyses were performed using GraphPad Prism 9.0 software (GraphPad Software Inc., La Jolla, CA). Data are presented as mean ± standard error of the mean (SEM). Nonparametric one-way ANOVA comparison was used to compare the control group with ARSACS group for each type of neurons. Tests having a *p* value above 0.05 were determined statistically different.

## 3. Results

### 3.1. Differentiation of iPSCs from ARSACS Patients into MNs

**Figure 1:**
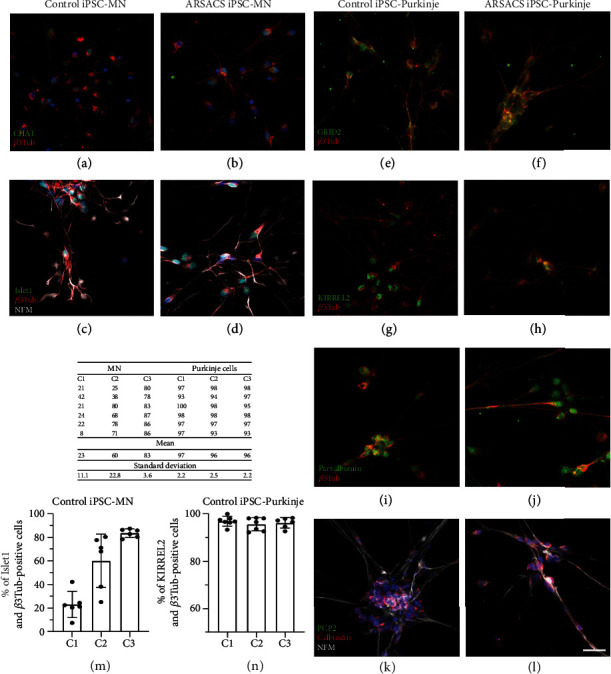
Characterization by immunofluorescence of MNs and Purkinje cells differentiated from healthy or ARSACS iPSCs cultured in 2D. MNs and Purkinje cells differentiated from iPSCs were seeded on poly-D-lysine coated wells and differentiated until days 13 and 14, respectively. MNs were characterized by immunofluorescent staining using the CHAT (a, b) (in green) and Islet-1 (c, d) (in green) specific MN markers and the neuronal markers NFM (c, d) (in white) and *β*3-tubulin (a–d) (in red). Purkinje cells were stained with neuronal markers *β*3-tubulin (e–l) (in red) and NFM (k, l) (in white) and with Purkinje cell-specific markers GRID2 (e, f) (in green), KIRREL2 (g, h) (in green), parvalbumin (i, j) (in green), PCP2 (k, l) (in green), and calbindin (k, l) (in red). Purity was assessed by immunofluorescence and counting MNs stained with Islet and *β*3-tubulin (m), and Purkinje cells were stained with KIRREL2 and *β*3-tubulin (n) (see raw data in the table). Nuclei in (a)–(d) and (k) and (l) were stained in blue with DAPI. Scale bar in (l): 20 *μ*M.

**Figure 2:**
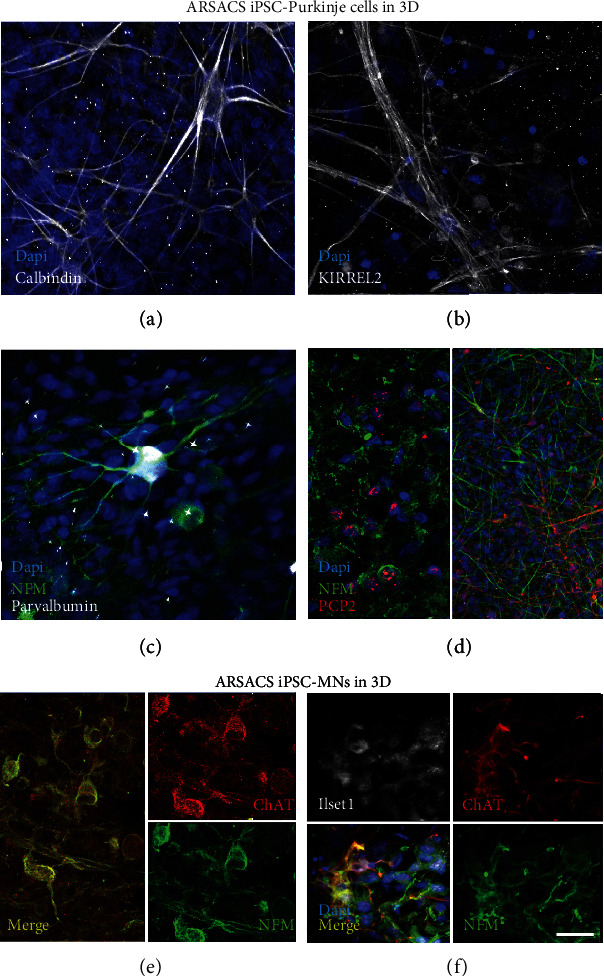
Immunofluorescence characterization of MNs and Purkinje cells differentiated from ARSACS iPSCs cultured in 3D. ARSACS iPSC-differentiated Purkinje cells were cultured on sponges containing fibroblasts for 53 days (a–d). ARSACS iPSC-differentiated MNs were cultured on sponges populated with fibroblasts and ARSACS iPSC-derived Schwann cells for 74 days (e, f). The 3D models were imaged by immunofluorescence through a view from above after staining of cells with the neuronal marker NFM (c, d) in green and with the specific markers calbindin (a), KIRREL2 (b), and parvalbumin (c) in white, PCP2 (d) in red for Purkinje cells, NFM in green, ChAT (e, f) in red, and Islet1 (f) in white for MNs. These results are representative of the three ARSACS cell populations. Nuclei were stained in blue with DAPI. Scale bar in (f): 20 *μ*m.

iPS cells derived from 3 ARSACS patients and 3 healthy individuals were differentiated into MNs during 13 days for 2D culture in 12-well plates coated with poly-D-lysine. MN maturity and identity was assessed by immunofluorescence staining with general neuronal cell markers such as neurofilament M (NFM; 160 kDa) and *β*3-tubulin and markers specific to MNs through Islet1 and ChAT. Both ARSACS and control iPSC-derived MNs expressed all these markers, demonstrating the efficacy of our differentiation protocol (Figures 1(a)–1(d)). No difference was observed in the expression of these markers between MNs differentiated from ARSACS iPSCs compared to control iPSCs in 2D culture. The efficiency of iPSC differentiation into MN was assessed on control iPSC lines by counting the proportion of cells coexpressing Islet1 and *β*3-tubulin (Figure 1(m)). The C3 cell line gave a better purity of 83% ± 3.6, compared to the C2 cell line (60% ± 22.7) and C1 (23% ± 11). When cultured in a 3D environment for 74 days in total, MNs coexpressed NFM and ChAT (Figure 2(e)) as well as Islet1 with NFM (Figure 2(f)) as observed in 2D culture.

### 3.2. Differentiation of iPSCs from ARSACS Patients into Purkinje Cells

The same ARSACS and control-derived iPSCs were also differentiated into Purkinje cells using poly-D-lysine coated 24 well plates for 14 days in 2D culture. At this stage, differentiated Purkinje cells displayed an elongated bipolar morphology. Cell identity and maturity were assessed by immunofluorescence staining of general neuronal markers using *β*3-tubulin or NFM, as well as specific Purkinje cell markers like GRID2, KIRREL2, parvalbumin, or calbindin (Figures 1(e)–1(l)). These markers were mostly expressed in the cell body, except for GRID2 that was also detected along some dendrites. Moreover, these cells did not express PCP2 at this early differentiation stage (Figures 1(k) and 1(l)). No difference was observed in the expression of these markers between Purkinje cells differentiated from ARSACS and control iPSCs in 2D culture.

The purity of Purkinje cells following iPSC differentiation was assessed by immunofluorescence analysis through counting the proportion of KIRREL2 and *β*3-tubulin coexpression in the cells. After 14 days of differentiation, more than 92% of iPSCs expressed both markers (Figure 1(n)).

A major limitation of growing Purkinje cells on plastic was their spontaneous detachment between day 15 and day 20 of in vitro differentiation, before reaching full maturation. To solve this problem, Purkinje cells were differentiated 11 days on plastic (before the neurites were formed) and transferred on a 3D substrate to complete the needed differentiation period for an additional 6 weeks. Immunofluorescence analysis showed that differentiated Purkinje cells expressed the neuronal marker NFM (Figures 2(c) and 2(d)) as well as known Purkinje cell markers calbindin (Figure 2(a)), KIRREL2 (Figure 2(b)), parvalbumin (Figure 2(c)), and PCP2 (Figure 2(d)). Moreover, some of these cells displayed a more stellar morphology with multiple dendrites (Figures 2(a) and 2(c)). Of note, these specific Purkinje cell markers were found to be homogeneously distributed and mostly detected along dendrites in long-term 3D cultures, whereas mainly detected in the cell body in 2D culture after 14 days of differentiation. In addition, PCP2 was not expressed at an earlier stage of differentiation in the 2D culture but was detected in the 3D model after 53 days of differentiation (Figures 1(k) and 1(l) vs. Figure 2(d)).

### 3.3. ARSACS Purkinje Cells and MNs Showed Decreased Levels of Sacsin but Not of DRP1

**Figure 3:**
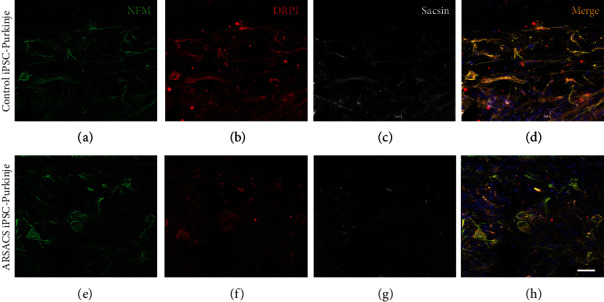
Immunofluorescence assessment of sacsin and DRP1 expression in Purkinje cells differentiated from healthy or ARSACS iPSCs cultured in 3D. iPSCs were seeded at a density of 285,000 cells/cm^2^ onto the sponge containing fibroblasts for a total in vitro differentiation of 53 days. The 3D model was imaged with a view from above after staining of cells for NFM in green (a, e, d, h), DRP1 in red (b, f, d, h), and sacsin in white (c, g, d, h). Nuclei were stained in blue with DAPI (d, h). Scale bar in (h): 20 *μ*m.

**Figure 4:**
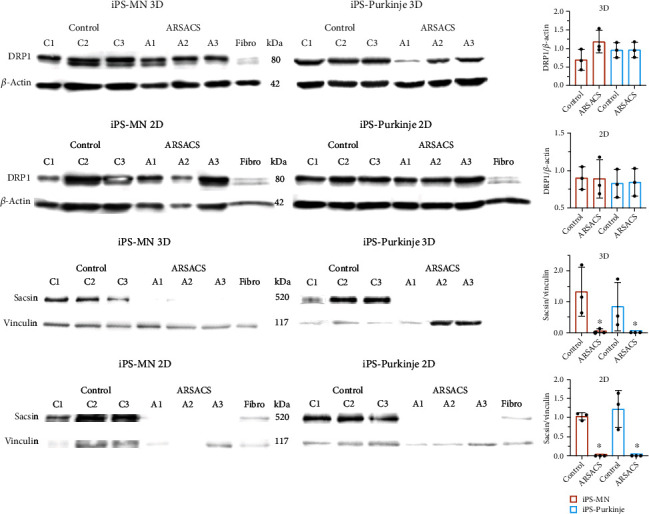
Assessment by Western blot analysis of DRP1 and sacsin expression levels in MNs and Purkinje cells differentiated from healthy and ARSACS iPSCs cultured in 2D and 3D. Expression levels of DRP1 and sacsin were assessed by Western blot analysis on MNs and Purkinje cells differentiated from 3 healthy control iPSCs (C1, C2, C3) and 3 ARSACS iPSCs (A1, A2, A3) cultured in 2D and 3D, as well as on fibroblasts. DRP1 and sacsin were, respectively, normalized against actin and vinculin to control for equal loading quantification. A statistical analysis was performed using 1-way ANOVA nonparametric test, ^∗^*p* < 0.0005 (*N* = 3).

The expression of DRP1 and sacsin proteins expressed by ARSACS and control Purkinje cells differentiated from iPSCs, cultured for 53 days in vitro on the 3D substrate, was assessed by immunofluorescence (Figure 3). Both DRP1 and sacsin expressions seemed less intense in the ARSACS-derived Purkinje cells (Figures 3(a)–3(d) versus Figures 3(e)–3(h)). Semiquantitative measurements of DRP1 and sacsin expressions were then performed by Western blot analysis using whole protein extracts collected from ARSACS and control Purkinje cells differentiated from iPSCs (Figure 4 and Figure S1 in Supplementary materials). A significant decrease of sacsin expression was detected in ARSACS iPSC-derived Purkinje cells (*N* = 3, *p* < 0.0005). This was measured both in cells differentiated 14 days in 2D culture as well as for cells differentiated in 3D collagen sponges for a total of 53 days. Note that healthy fibroblast expression of sacsin and DRP1 was found to be very low when compared to iPSC-derived Purkinje cells, which may therefore not interfere with the measurement of the total expression of these proteins in 3D experiments where both cell types are cocultured in the sponges.

Conversely, the expression of DRP1 was found to be similar in control versus Purkinje cells differentiated from ARSACS-derived iPSCs independent of the 2D or 3D culture methods. Similar results, i.e., a reduced sacsin expression level (*N* = 3, *p* < 0.0005) but comparable expression of DRP1, were observed in MNs differentiated from ARSACS-derived iPSCs cultured in 2D for 13 days as well as in 3D for 74 days. In this last condition, MNs were cocultured with healthy fibroblasts but with ARSACS iPSC-derived Schwann cells in the ARSACS model (to promote axonal migration as previously described [39]).

### 3.4. Neurofilament M Accumulation Was Observed in Purkinje Cells and MN Neurites

**Figure 5:**
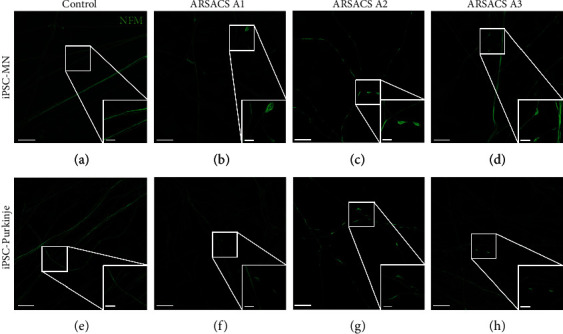
Formation of NFM aggregates in neurites from MNs and Purkinje cells differentiated from ARSACS iPSCs cultured in 3D. Cells were seeded onto the sponge containing fibroblasts during a total in vitro differentiation of 53 days for Purkinje cells and 74 days for MNs. The 3D model was imaged with a view from above after staining of cells for NFM in green (a–h). Neurites elongated from MNs and Purkinje cells differentiated from the healthy control into MNs (a) or Purkinje cells (e) and were compared to 3 ARSACS iPSC cell lines (A1, A2, A3) differentiated into MNs (b–d) or Purkinje cells (f–h). NFM accumulation was highlighted in white squares enlarged in the bottom right corner of (a)–(h). Scale bars: 20 *μ*m.

The accumulation of NFM (Figure 5) was further analyzed by immunofluorescence after differentiation of iPSC-derived Purkinje cells and iPSC-derived MNs on 3D collagen sponges. Respectively after 53 and 74 days of maturation, Purkinje cells (Figures 5(f)–5(h)) and MNs (Figures 5(b)–5(d)), differentiated from patient iPSCs, showed NFM aggregates, characteristic to ARSACS, along their neurites in contrast to the control (Figures 5(a) and 5(e)) [40].

## 4. Discussion

We have developed in this study simplified and easily reproducible protocols for the generation of MNs and Purkinje cells differentiated from ARSACS patient-derived iPSCs, which successfully recapitulated the early pathological signature of the disease. Extracting living neurons from the cerebellum and other CSN tissues remains very difficult to achieve from postmortem human pathological tissue [4]. To overcome this drawback, several protocols have been developed to differentiate iPSCs into Purkinje cells to generate cerebellum organoids [41–43]. However, in the vast majority of these protocols, the coculture of Purkinje cells with animal cerebellar cells or on organotypic cerebellar slices are needed to facilitate the iPSC differentiation [14, 15, 17].

Here, the goal of this study was to develop a culture system entirely made of human cells, using Purkinje cells or MNs differentiated from iPSCs obtained from ARSACS patients.

Although it was previously showed that it was possible to generate an iPSC line from ARSACS patient dermal fibroblasts, these patient-derived iPSCs were not differentiated into specific neuronal cells [44, 45].

We showed for the first time that it was possible to differentiate ARSACS-iPSCs into MNs and Purkinje cells using the 3D culture system we developed, as well as to detect some characteristic ARSACS pathological features such as abnormal accumulation of NFM along the neurites. Using collagen sponges populated with dermal fibroblasts as a 3D substrate, which is known to promote long-term survival and neurite elongation of MNs [39] and to reproduce pathological features observed in other brain diseases such as neurofibromatosis [46] and amyotrophic lateral sclerosis [47, 48], it was possible to enable long term (>53 days) of Purkinje cells and to promote abundant elaborated dendritic ramifications. To better mimic ARSACS cellular microenvironment around MNs, iPSC-derived Schwann cells were also cocultured with iPSC-MNs within the 3D substrate. Such condition allowing the coculture of Schwann cells with iPSC-derived MNs was previously shown to be essential to enhance axonal migration and to promote myelin sheath formation around neurites [39, 48].

We also showed that sacsin expression was greatly reduced in both iPSC-MNs and Purkinje cells derived from ARSACS patients, similarly to what it is already described in the literature in patients, ARSACS mice [9, 11], and other *in vivo* studies [7]. We did not observe however misexpression of DRP1 in our model, previously shown to be slightly downregulated following loss of sacsin expression.

Finally, we showed for the first time NFM aggregate formation in ARSACS patient-derived Purkinje cell and MN neurites, as several studies confirmed when the sacsin protein is altered in mouse or human nonneuronal cells [7, 13, 40, 49]. However, this effect was only detected after long-term maturation of these cells in our 3D culture conditions. Moreover, we did not observed accumulation of neurofilaments as seen in mouse *Sacs*^−/−^ motor neurons and human ARSACS brain [7]. This may be due to a differentiation period of MN and Purkinje cells that was too short in vitro to allow this characteristic to appear.

This in vitro model, shown to recapitulate important ARSACS pathological features, could serve as a tool to better understand the disease mechanism and to test new therapeutic molecules or new strategies against ARSACS.

## 5. Conclusion

We present a new in vitro culture system allowing the differentiation of ARSACS patient-derived iPSCs into MNs and Purkinje cells and long-term coculture of these two neuronal types. In future studies, it will be interesting to complexify the model by incorporating other subpopulations of cerebellar cells such as granule cells, oligodendrocytes, or Bergmann glial cells.

## Data Availability

Source data are provided in this paper. All relevant data are available from the authors upon reasonable request.

## Supplementary Materials

Supplementary Materials Supplementary Table 1: indications of the sex, age, and genotype of the parental cell lines used from ARSACS patients. Supplementary Figure S1: original Western blots used in Figure 4.
