# Supplementary material for: In Vitro Characterization of Motor Neurons and Purkinje Cells Differentiated from Induced Pluripotent Stem Cells Generated from Patients with Autosomal Recessive Spastic Ataxia of Charlevoix-Saguenay
Source: Stem Cells Int. 2023 Apr 15;2023:1496597. doi: 10.1155/2023/1496597 (PMC10122584; doi:10.1155/2023/1496597)
Supplement: Supplementary Materials — Supplementary Table 1: indications of the sex, age, and genotype of the parental cell lines used from ARSACS patients. Supplementary Figure S1: original Western blots used in Figure 4. [file 1496597.f1.pdf]

Supplementary materials

In vitro characterization of motor neurons and Purkinje cells differentiated from induced pluripotent stem cells generated from patients with autosomal recessive spastic ataxia of Charlevoix-Saguenay

Aurélie Louit[1‡], Marie-Josée Beaudet[1‡], Mathieu Blais[1], François Gros-Louis[1,2], Nicolas Dupré[1,3], François Berthod[1,2*]

[1]LOEX, Centre de recherche du CHU de Québec-Université Laval, Quebec City, Quebec, Canada
[2]Department of Surgery, Faculty of Medicine, Université Laval, Quebec City, Quebec, Canada
[3]Department of Medicine, Faculty of Medicine, Université Laval, Quebec City, Quebec, Canada

**ARSACS Cell line**	**FARSF56-7**	**FARSM50-5**	**SK.01AiPS**
**Abbreviation**	A1	A2	A3
**Sex**	Female	Male	Female
**Age**	56	50	35
**Genotype**	Homozygous c.8844delT	Homozygous c.8844delT	Homozygous c.9284dupC

Supplementary Table 1: Indications of the sex, age and genotype of the parental cell lines used from ARSACS patients.

Supplementary figure S1: Original Western blots used in figure 4.

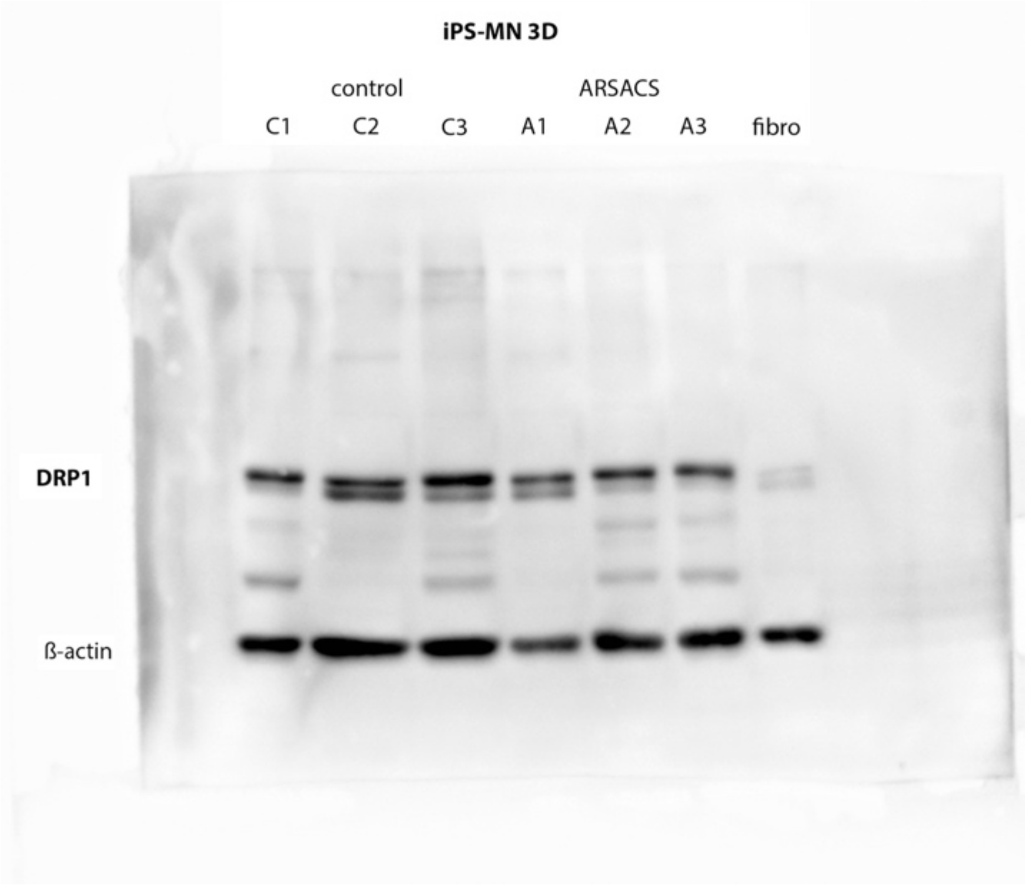

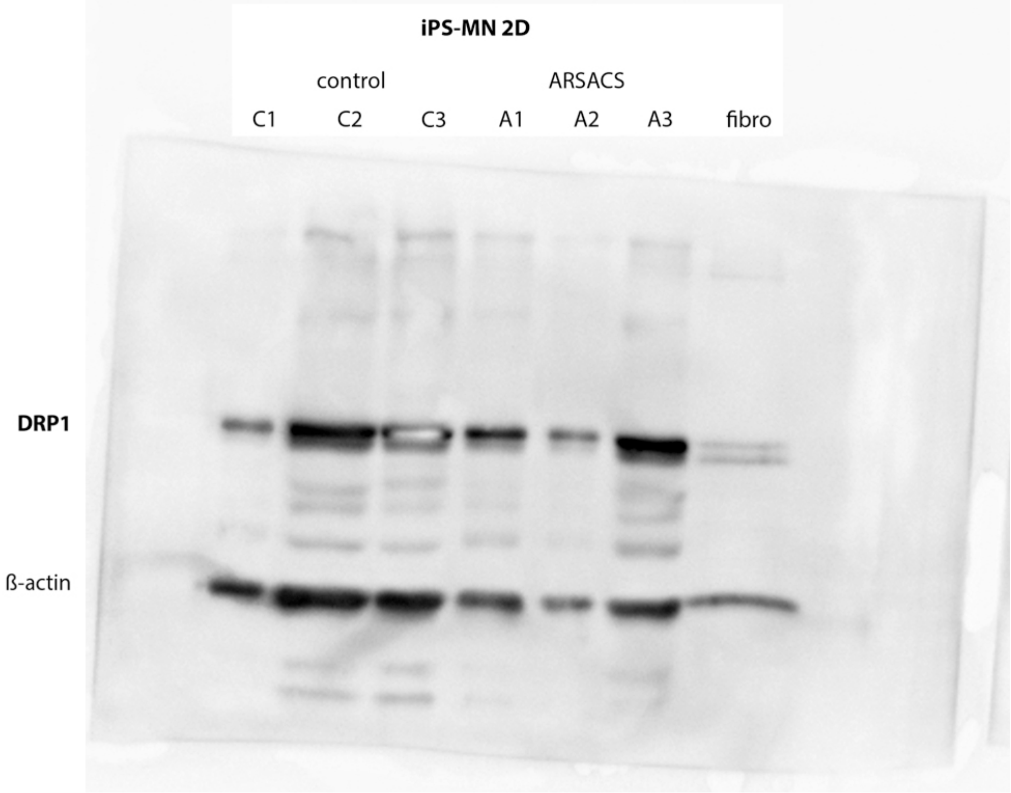

iPS-MN 3D

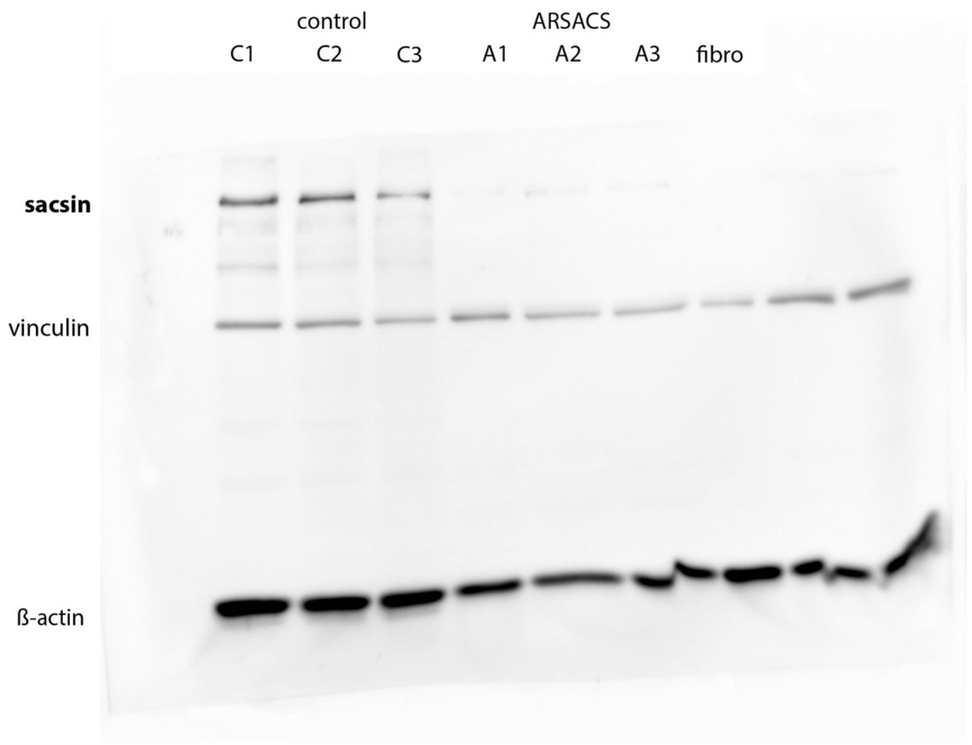

iPS-MN 2D

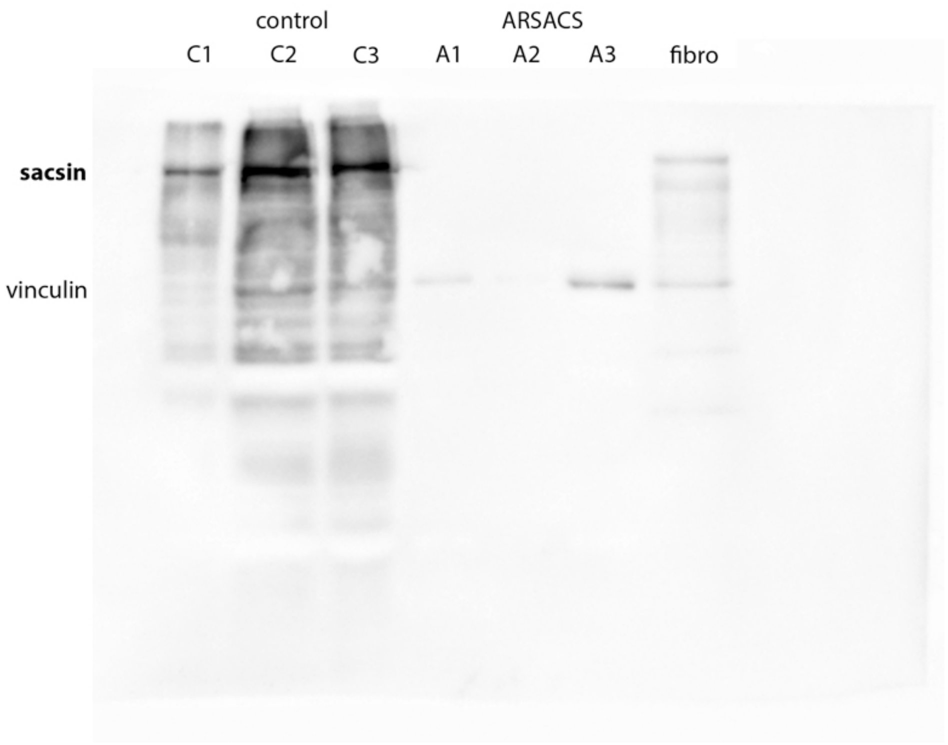

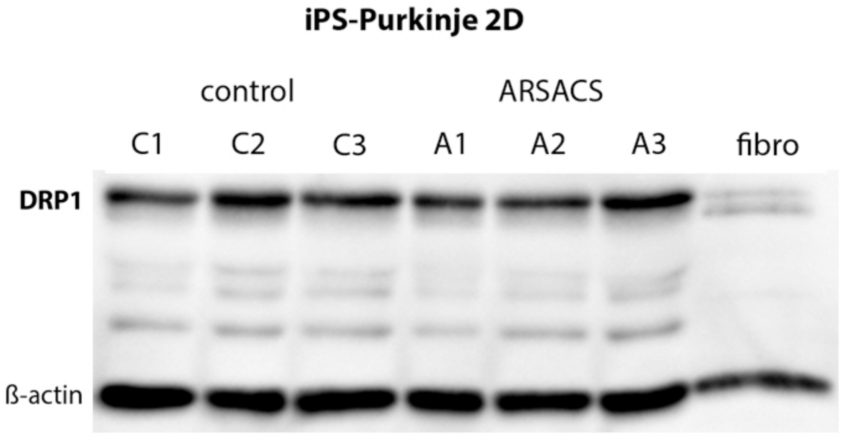
iPS-Purkinje 2D
control
ARSACS
C1
C2
C3
A1
A2
A3
fibro
DRP1
ß-actin

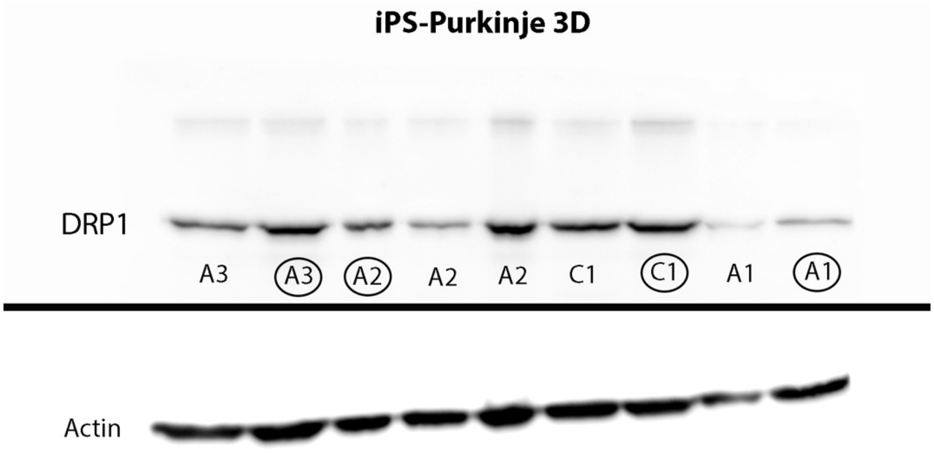
iPS-Purkinje 3D
DRP1
A3
A3
A2
A2
A2
C1
C1
A1
A1
Actin

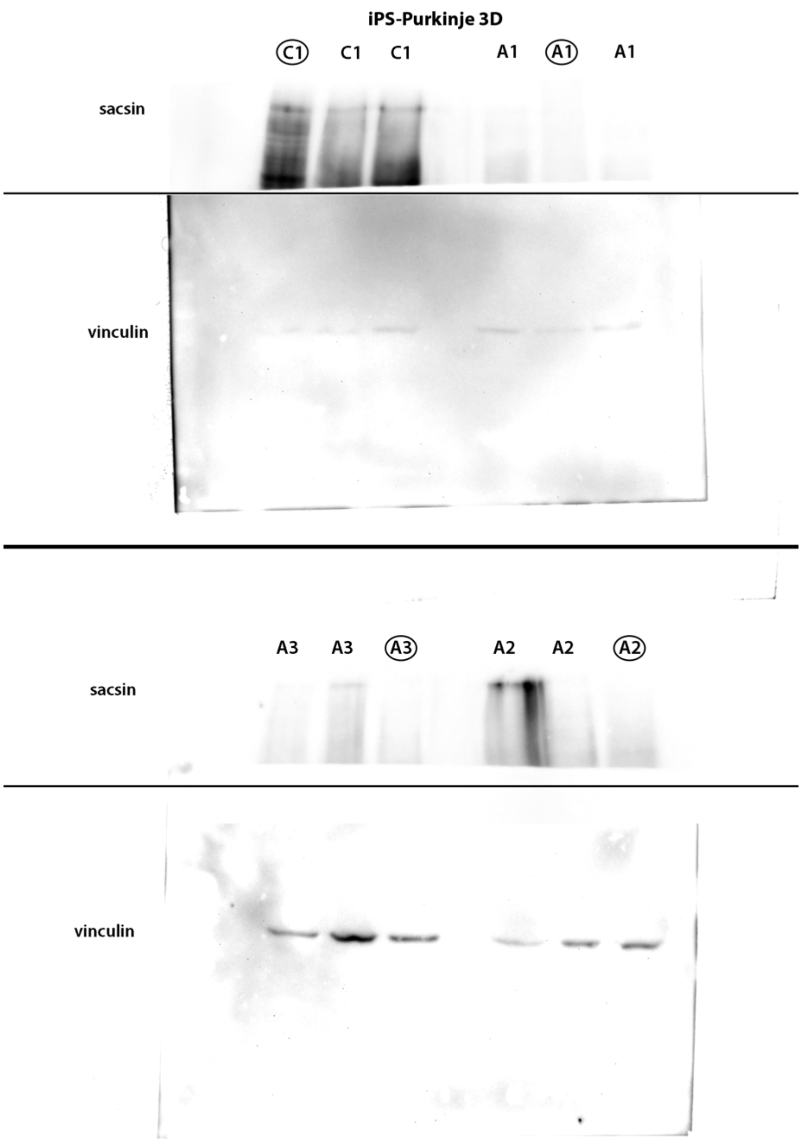
iPS-Purkinje 3D
C1 C1 C1 A1 A1 A1
sacsin
vinculin
A3 A3 A3 A2 A2 A2
sacsin
vinculin

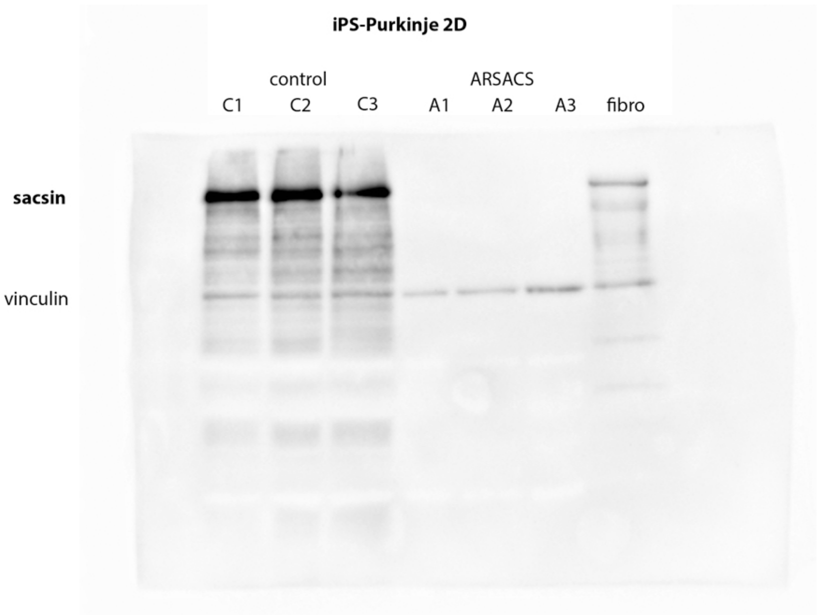
iPS-Purkinje 2D
control
ARSACS
C1 C2 C3 A1 A2 A3 fibro
sacsin
vinculin
